# Impact of new guidelines for microbiological sampling on antibiotic prescribing patterns in general practice in the North Denmark Region: a retrospective pre-post observational study

**DOI:** 10.1080/02813432.2026.2726274

**Published:** 2026-09-24

**Authors:** Pernille Thy Christensen, Sofie Byskov, Alina Zalounina Falborg, Niels Kristian Skibsted, David Fuglsang-Damgaard, Malene Plejdrup Hansen

**Affiliations:** a Department of Clinical Medicine, Aalborg University, Aalborg, Denmark; b Center for General Practice, Department of Clinical Medicine, Aalborg University, Aalborg, Denmark; c Research Unit for General Practice, Aarhus, Denmark; d Quality Unit for General Practice, Aalborg, Denmark; e Department of Clinical Microbiology, Aalborg University, Aalborg, Denmark

**Keywords:** General practice, diagnostic stewardship, antibiotic use, antibiotic stewardship

## Abstract

**Background:**

In April 2024, the Department of Clinical Microbiology at Aalborg University Hospital introduced new guidelines for general practitioners in the North Denmark Region, limiting microbiological culture and susceptibility testing of wound, skin, eye, ear, and throat infections to recurrent cases or treatment failure. While intended to reduce unnecessary testing and optimize resource use, the effect on antibiotic prescribing patterns remains unclear.

**Objective:**

To characterize and explore the development in requested microbiological cultures and susceptibility testing and antibiotic prescribing patterns in general practice in the North Denmark Region before and after the introduction of the new guidelines. Antibiotic prescribing was also compared with the Central Denmark Region.

**Materials and methods:**

This retrospective pre-post observational study included data from the Department of Clinical Microbiology on submitted and rejected samples from general practices in the North Denmark Region. Furthermore, number of redeemed antibiotic prescriptions from general practices in both the North and Central Denmark Regions were extracted from the Ordiprax + database (available from the Danish Health Data Authority). Two time periods were compared: pre-guideline (April 2023–March 2024) and post-guideline (April 2024–March 2025). Incidence rates and incidence rate ratios were calculated to assess changes in prescribing patterns.

**Results:**

Submitted samples decreased by 66.4% following guideline implementation. Antibiotic prescribing in the North Denmark Region decreased slightly (–2.4%), with minor changes in prescribing patterns. Similar changes were observed in the Central Denmark Region.

**Conclusion:**

The guidelines were effectively adopted but had limited impact on antibiotic prescribing in general practice. Further research should assess clinical outcomes, treatment quality, and long-term effects on antimicrobial resistance.

## Introduction

Microbiological laboratories play a central role in infection diagnostics, but routine testing also requires substantial use of consumables and laboratory resources [1]. Reducing the use of materials for routine microbiological analyses is expected to lower environmental impact, generate economic benefits, and free up personnel time and resources that can be reallocated to enhance the quality of the analyses [2,3]. Evidence suggest that the bacterial aetiology of superficial wound, skin, eye and ear infections is generally well characterised [4,5]. Given that the aetiology in most cases is well known and the prevalence of antimicrobial resistance (AMR) among predominant pathogens is low [6], treatment can often be initiated empirically. In this context, microbiological test results may have limited impact on clinical management. From April 20th, 2024 the Department of Clinical Microbiology at Aalborg University Hospital introduced new guidelines regulating cultures and susceptibility testing from general practices in the North Denmark Region. For suspected infections in wounds, skin, eyes, ears, and throat, general practitioners (GPs) must now answer two questions when requesting a culture:

1. Is this a recurrent infection?
2. Has there been treatment failure despite appropriate therapy?

Cultures are only performed if at least one of these criteria is fulfilled [6]. While the guidelines help minimize unnecessary testing, they also reduce access to microbiological confirmation before treatment is initiated. Although clinical assessment is often sufficient to diagnose common bacterial infections, culture results can provide additional diagnostic certainty and help guide antibiotic treatment, particularly in more uncertain cases. These recommendations should be viewed alongside the Danish Health Authority guideline, which states that antibiotic treatment should only be initiated when the overall clinical assessment together with any relevant diagnostic investigation makes a bacterial cause sufficiently likely [7,8]. A concern might be that restricted testing may increase diagnostic uncertainty and lead to more antibiotic prescriptions being issued and/or greater use of broader-spectrum antibiotics. This issue is particularly important given the global threat of AMR. According to World Health Organization’s (WHO) Global Action Plan on AMR, resistant pathogens caused an estimated 1.27 million deaths in 2019 [9]. Excessive and inappropriate antibiotic use is a key driver of resistance, which threatens the ability to treat infections and safely perform procedures such as chemotherapy and major surgery [10]. Reducing unnecessary antibiotic prescriptions is therefore essential to mitigate the development of resistance. On the other hand, sometimes the clinical presentation of a bacterial infection is clear, and awaiting microbiological results before initiating antibiotic treatment, would be inappropriate.

Despite these concerns, it has not yet been systematically examined how the new guidelines for cultures and susceptibility testing have impacted the antibiotic prescribing patterns in general practices. There is limited knowledge on how restricting cultures and susceptibility testing has affected the volume and type of antibiotics prescribed. The aim of this study was to characterize and explore the development in requested microbiological cultures and susceptibility testing and antibiotic prescribing patterns in general practice in the North Denmark Region before and after the introduction of the new guidelines. In addition, a comparison between the antibiotic prescribing patterns in the North Denmark Region and the Central Denmark Region was performed.

## Materials and methods

### Study design and setting

This retrospective pre-post observational study used data from the Department of Clinical Microbiology at Aalborg University Hospital, serving the North Denmark Region population (approximately 590,000 inhabitants). The Department of Clinical Microbiology provides services for all hospitals, general practices, and local specialists within the region.

The study also includes data on antibiotic prescriptions from general practice in both the North Denmark Region and the Central Denmark Region. The data were divided into two periods corresponding to the introduction of new guidelines: a pre-guideline period from April 1st, 2023 to March 31st, 2024, and a post-guideline period from April 1st, 2024 to March 31st, 2025. For the Central Denmark Region, the same time periods were used to allow for comparison, as this region did not implement the new guidelines during the study period. The Central Denmark Region was chosen as reference region due to its comparability in terms of healthcare organisation, including sampling strategies and clinical guidelines, as well as to minimise potential bias from temporal variation and epidemic timing.

### Data sources and variables

#### Data from Department of Clinical Microbiology

The dataset from the Department of Clinical Microbiology was extracted from the laboratory information system wwLab (Mylab, Tampere, Finland) and comprised the number of microbiological samples from wounds, skin, eyes, ears, and throat submitted by GPs in the North Denmark Region, as well as the number of cultures and susceptibility tests performed, and samples rejected. Samples were rejected according to standard laboratory pre-analytical criteria, including incorrect patient identification, insufficient sample material, inappropriate sampling or transport conditions, and incomplete requisition information. The analysis included the following sampling labels: wound (‘Sårpodning’ and related labels), skin (‘Hudpodning’, ‘Pustel-podning’), eye (‘Øjenpodning’, ‘Pus – podning øje’), ear (‘Ørepodning’, ‘Ørepodning otitis media’), throat (‘Svælgpodning’, ‘Podning – peritonsillær’) and unspecified (‘Podning’).

#### Data from the North Denmark Region and Central Denmark Region

The data from the North Denmark Region and Central Denmark Region were extracted from the Ordiprax + database (Danish Health Data Authority) on October 20th, 2025 [11]. The dataset included information about number and type of antibiotics redeemed in all general practices located in each of the Regions.

### Statistical analysis

Data on microbiological cultures and susceptibility testing were presented as the monthly number of cultures performed/samples rejected, respectively. Furthermore, data on antibiotic prescription was presented as (a) the monthly number of prescriptions, total and stratified by type of antibiotic, and (b) the monthly number of prescriptions per 1,000 persons, i.e. the incidence rate (IR). Incidence rate ratios (IRR) with 95% confidence intervals (95% CI) were calculated to compare prescribing patterns: (a) between the two periods within each region, and (b) between the two regions within each period. Differences between the two regions in changes in prescribing patterns from the pre-guideline to the post-guideline period were assessed using the ratio of IRRs. The analyses were performed in RStudio [12].

### Ethics and data protection

This study was registered at the North Denmark Region (ID F2025-077). According to Danish law, this type of study does not require ethical approval. All data were treated confidentially in accordance with the Danish Data Protection Act.

### AI-based tools and technologies

ChatGPT was used solely for linguistic and grammatical assistance and did not contribute to the scientific content, data interpretation, or conclusion.

## Results

### Microbiological cultures and susceptibility testing in the North Denmark Region

A total of 31,931 and 10,695 samples were submitted to the Department of Clinical Microbiology for analysis during the pre-guideline and post-guideline periods, respectively (Table 1). On average, 1,770 fewer cultures were performed per month in the post-guideline period compared to the pre-guideline period, representing a reduction of 66.4%. At the same time, the number of rejected samples increased substantially: during the pre-guideline period, 114 samples were rejected in total, whereas 1,554 samples were rejected after the guidelines were introduced (Table 2).

**Table 1.** Monthly number of microbiological cultures in the pre- and post-guideline period in North Denmark Region.

Month	Pre-guideline (2023–2024)	Post-guideline (2024–2025)	Difference	Difference (%)
April	2,522	2,274	−248	−9.8
May	2,841	1,153	−1,688	−59.5
June	3,009	891	−2,118	−70.4
July	2,484	819	−1,665	−67.0
August	2,838	918	−1,920	−67.7
September	2,851	836	−2,015	−70.7
October	2,649	821	−1,828	−69.0
November	2,769	674	−2,095	−75.7
December	2,268	540	–1,728	−76.2
January	2,673	651	−2,022	–75.7
February	2,537	540	−1,997	−78.7
March	2,490	578	−1,912	−76.8
Total	**31,931**	**10,695**		
Total difference			−**21,236**	
Mean difference			−**1,769.7**	**−66,4**

**Table 2.** Monthly number of rejected samples in the pre- and post-guideline period in North Denmark Region.

Month	Pre-guideline (2023–2024)	Post-guideline (2024–2025)	Difference
April	10	291	281
May	8	304	296
June	7	156	149
July	10	136	126
August	3	129	126
September	7	109	102
October	12	90	78
November	21	82	61
December	14	68	54
January	7	75	68
February	7	65	58
March	8	49	41
Total	**114**	**1,554**	
Mean difference			**120**

Figure 1 illustrates the development in the number of samples submitted to the Department of Clinical Microbiology from April 1st, 2023 to March 31st, 2025, categorised by sampling location; wounds, skin, eyes, ears, and throat, and ‘unspecified’, as well as the number of rejected samples. The figure demonstrates a marked decrease in the number of submitted samples between April and May 2024, corresponding with the time of introduction of the new guidelines for microbiological cultures and susceptibility testing. Furthermore, an increase in the number of rejected samples from April 2024 onwards is shown, followed by a gradual monthly decline until March 2025.

**Figure 1.**
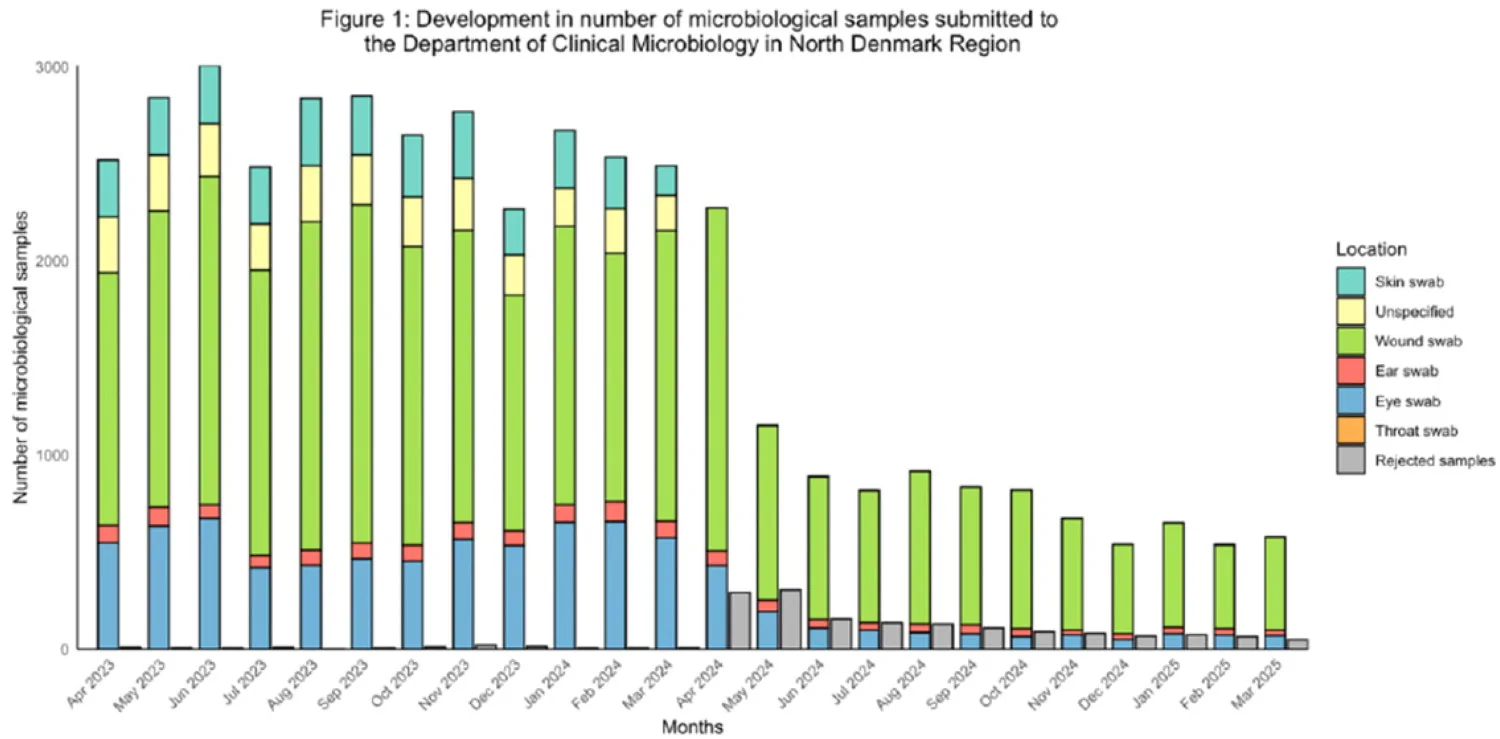
Development in number of microbiological samples submitted to the Department of Clinical Microbiology in North Denmark Region. *Note:* As of April 2024, samples previously labelled as skin swabs or ‘unspecified’ were reclassified to be included under wound swabs.

### Antibiotic prescribing in the North Denmark Region

The total number of redeemed antibiotic prescriptions from general practice in the North Denmark Region decreased from 196,931 prescriptions in the pre-guideline period to 191,747 prescriptions in the post-guideline period corresponding to a decrease of 2.4% (Table 3). Furthermore, monthly variation with some months exhibiting increases and others decreases in prescription numbers could be observed.

**Table 3.** Monthly number of antibiotic prescriptions in the pre- and post-guideline period in North Denmark Region.

Month	Pre-guideline (2023–2024)	Post-guideline (2024–2025)	Diff (%)
April	15522	17018	1496 (9.6)
May	16164	16061	−103 (−0.6)
June	16150	14273	−1877 (−11.6)
July	14404	15177	773 (5.4)
August	16106	15637	−469 (−2.9)
September	16350	15444	−906 (−5.5)
October	15948	16655	707 (4.4)
November	17222	16178	−1044 (−6.1)
December	17478	15167	−2311 (−13.2)
January	18261	17160	−1101 (−6.0)
February	17106	16114	−992 (−5.8)
March	16220	16863	643 (4.0)
Total	**196931**	**191747**	
Mean difference			**−432 (−2.4)**

### Antibiotic prescribing patterns in general practices in the North and Central Denmark

#### Regions

In both regions, the rate of antibiotic prescriptions decreased from the pre-guideline to the post-guideline period, with IRRs of 0.975 (95% CI: 0.969–0.981) in the North Denmark Region and 0.963 (95% CI: 0.959–0.967) in the Central Denmark Region (Table 4). This reduction was statistically significantly larger in the Central Denmark Region than in the North Denmark Region (ratio of IRRs: 0.987, 95% CI: 0.980–0.995).

**Table 4.** Incidence rates (IR) and incidence rate ratios (IRR) of antibiotic prescriptions per 1,000 persons in North Denmark Region (pre- vs post-guideline) and Central Denmark Region (same periods as reference).

Group of antibiotics	North Denmark Region Pre-guideline IR (2023–2024)	North Denmark Region Post-guideline IR (2024–2025)	North Denmark Region IRR (95% CI)	Central Denmark Region IR (2023–2024)	Central Denmark Region IR (2024–2025)	Central Denmark Region: IRR (95% CI)
Beta-lactamase sensitive penicillins	73.5	71.5	0.97 (0.95–0.98)	67.7	66.9	0.98 (0.97–0.99)
Beta-lactamase resistant penicillins	32.5	31.1	0.95 (0.93–0.97)	30.4	31.0	1.01 (1.00–1.03)
Penicillins with extended spectrum	95.0	95.5	1.00 (0.99–1.01)	86.9	85.4	0.98 (0.97–0.99)
Macrolides and lincosamides	21.9	24.9	1.13 (1.11–1.16)	23.3	27.3	1.17 (1.15–1.18)
Cephalosporins	0.06	0.05	0.80 (0.50–1.27)	0.06	0.05	0.82 (0.60–1.12)
Flouroquinolones	6.0	5.4	0.90 (0.86–0.94)	4.8	4.0	0.83 (0.80–0.86)
Metronidazole	5.7	4.4	0.77 (0.73–0.81)	4.7	3.9	0.83 (0.80–0.86)
Others	33.6	33.8	1.00 (0.98–1.02)	32.4	28.6	0.88 (0.87–0.89)
Topical ear antibiotics	17.19	18.3	1.07 (1.04–1.09)	18.3	18.4	1.00 (0.98–1.02)
Topical eye antibiotics	37.8	30.6	0.80 (0.70–0.82)	36.4	28.9	0.79 (0.78–0.80)
Topical skin antibiotics	9.5	8.8	0.93 (0.89–0.96)	16.7	15.3	0.91 (0.89–0.93)
Overall	**333.16**	**324.90**	**0.975 (0.969–0.981)**	**322.3**	**310.4**	**0.963 (0.959–0.967)**

Some changes were observed in the antibiotic prescribing patterns between pre- and post-guideline periods; however, similar changes were observed in both Regions (Table 4). For example, an increase in use of macrolides and lincosamides were noted with IRRs of 1.13 (95% CI: 1.11–1.16) and 1.17 (95% CI: 1.15–1.18) in the North and Central Denmark Regions, respectively. A reduced use of topical eye antibiotics was observed in both Regions (IRR= 0.80 (95% CI: 0.70–0.82) and 0.79 (95% CI: 0.78–0.80)), respectively. Also, the use of topical antibiotics for treatment of skin infections decreased (IRR= 0.93 (95% CI: 0.89–0.96) and 0.91 (95% CI: 0.89–0.93). In contrary to this, topical ear antibiotics was used more in the North Denmark Region, IRR = 1.07 (95% CI: 1.04–1.09) in the post-guideline period.

## Discussion

### Main findings

A significant decrease in the number of cultures performed (−66.4%) was observed in the North Denmark Region after the introduction of the new guidelines for cultures and susceptibility testing. Overall antibiotic use slightly decreased in the post-guideline period in the North Denmark Region (−2.4%) with some changes in the antibiotic prescribing patterns. Similar trends were observed in the Central Denmark Region with a slightly larger reduction in antibiotic prescribing in the post-guideline period.

### Strengths and limitations

A key strength of this study is the use of large datasets covering both antibiotic prescriptions and submitted samples for culture and susceptibility testing. Moreover, data on antibiotic prescriptions were obtained from a highly reliable national source, ensuring accuracy and completeness [11]. This study included data from both the North and Central Denmark Regions, enabling comparison of antibiotic use across regions.

This study has some limitations that should be noted. Firstly, it was decided to include April 2024 as part of the post-guideline period, even though the new guidelines were implemented during April 2024. It can be argued that an ‘embargo’ period (e.g. omitting April and May 2024) should have been included to allow for clinical uptake and adaptation to the new guidelines. As presented above (result section) the difference between number of cultures performed in April 2023 and April 2024 (−9.8%) was not as large as all other pre-post guideline comparisons (from −59.5% to −78.7%). Consequently, including April 2024 in the post-guideline period causes an underreporting of the *total reduction in cultures performed* one year after the guideline introduction. However, as the aim of this study was to characterize and explore the development in samples submitted and antibiotic prescribing patterns the authors believe that it is valuable to provide transparent data for each of the included months.

Secondly, the prescription data used reflect the total antibiotic use in the two regions across all types of infections treated, including those unlikely to be affected by the new guidelines. This broad measure may dilute any guideline-related changes in prescribing patterns and thus limit the ability to detect a true effect. Nevertheless, because overall antibiotic consumption remained largely stable during the study period, this limitation is unlikely to have substantially altered the conclusions. Furthermore, detailed information on the use of beta-lactamase sensitive penicillin or beta-lactamase resistant penicillin is available in Table 4. These antibiotics are preferred choices for treatment of skin and soft tissue infections and when comparing these specifically, no increase in usage was observed.

Thirdly, it should be noted that the pre-intervention period followed a period with an unusually high incidence of invasive and non-invasive group A streptococcal infections in Denmark. Although surveillance data suggest that the epidemic had largely subsided by May 2023, and that testing activity had returned towards usual levels, a residual influence on antibiotic prescribing, especially in the pre-guideline period, cannot be completely ruled out [13]

Finally, it cannot be determined with certainty that the samples submitted to the Department of Clinical Microbiology after the introduction of the new guidelines originated from patients who indeed did experience treatment failure or recurrence (as recommended in the new guidelines). The submissions may also reflect GPs with a strong desire for diagnostic certainty who therefore selected ‘yes’ to the relevant questions in the electronic requisition form.

### Findings in relation to other studies

In our study we found a decline in rejected samples submitted to the Department of Clinical Microbiology, suggesting that GPs in the North Denmark Region have adopted the new guidelines. While implementation of new recommendations in general practice is often met with variable acceptance [14,15], our findings indicate that the new guidelines were largely adhered to. In comparison, revised referral guidelines for lumbar spine magnetic resonance imaging (MRI) have proven more challenging to implement, as audits show that up to 65% of referrals are rejected due to insufficient indications, highlighting that compliance with restrictive measures can vary considerably [16,17].

This study examines the impact of the new guidelines for microbiological cultures and susceptibility testing which are based on the underlying assumption that empirical treatment is sufficient in most cases. This assumption is supported by a German study reporting that empirical therapy for infections in the ear, nose, and throat region is often sufficient [18]. Similarly, cultures from wounds may have limited diagnostic value, as open wounds are frequently colonized without indicating active infection [19]. Nevertheless, microbiological testing may remain valuable in selected cases, such as treatment failure or infections caused by resistant bacteria. However, in general practice, microbiological testing of wounds typically relies on swab sampling followed by semiquantitative culture. The diagnostic performance of this method is limited and may be influenced by variability in sampling, suggesting that culture findings alone may not reliably establish the presence or absence of an infection [19].

Previous research has examined how diagnostic testing may influence patient adherence to antibiotic treatment. For instance, an English study found that patients diagnosed with streptococcal throat infection demonstrated increased compliance with antibiotic treatment when a rapid antigen detection test had been performed [20]. Additionally, a qualitative study exploring patients’ experiences with rapid microbiological point-of-care tests found that using a diagnostic test could reduce reconsulting for the same illness [21]. These findings suggest that point-of-care testing may influence patient behaviour at the time of consultation. However, this effect may not be directly transferable to microbiological samples sent to external laboratories, where results are delayed and clinical decision-making has already taken place.

While diagnostic testing in general may strengthen patient confidence and improve treatment adherence, it also demands additional healthcare resources, and it remains uncertain whether increased diagnostic certainty translates into better clinical outcomes.

### Future research

Future research should investigate the clinical consequences of reduced microbiological testing, including its impact on patient outcomes such as recovery, complications and treatment adherence. Studies incorporating patient-level data could clarify the relationship between empirical treatment, diagnostic testing, and clinical effectiveness. In addition, there is limited knowledge on how the quality of care has changed following the introduction of the new guidelines. It remains unclear whether patients previously received optimal or suboptimal treatment, and whether changes in testing practices have influenced the appropriateness of antibiotic prescribing. Finally, qualitative studies exploring both GPs and patients’ experiences with the new guidelines could provide valuable insights into factors influencing guideline adherence and perceptions of diagnostic certainty. In addition, it would be relevant to explore variation in diagnostic testing practices between GPs and clinics, as well as differences in testing behaviour across clinical indications, patient characteristics, and organisational factors.
